# Mice lacking the cerebral cortex develop normal song: Insights into the foundations of vocal learning

**DOI:** 10.1038/srep08808

**Published:** 2015-03-06

**Authors:** Kurt Hammerschmidt, Gabriela Whelan, Gregor Eichele, Julia Fischer

**Affiliations:** 1 Cognitive Ethology Laboratory, German Primate Center, 37077 Göttingen, Germany; 2 Genes and Behavior Department, Max Planck Institute for Biophysical Chemistry, 37077 Göttingen, Germany

## Abstract

Mouse models play an increasingly important role in the identification and functional assessment of speech-associated genes, with a focus on genes involved in vocal production, and possibly vocal learning. Moreover, mice reportedly show direct projections from the cortex to brainstem vocal motor neurons, implying a degree of volitional control over vocal output. Yet, deaf mice did not reveal differences in call structures compared to their littermates, suggesting that auditory input is not a prerequisite for the development of species-specific sounds. To elucidate the importance of cortical structures for the development of mouse ultrasonic vocalizations (USVs) in more detail, we studied *Emx1-CRE*;*Esco2*^fl/fl^ mice, which lack the hippocampus and large parts of the cortex. We conducted acoustic analyses of the USVs of 28 pups during short-term isolation and 23 adult males during courtship encounters. We found no significant differences in the vocalizations of *Emx1-CRE*;*Esco2*^fl/fl^ mice, and only minor differences in call type usage in adult mice, compared to control littermates. Our findings question the notion that cortical structures are necessary for the production of mouse USVs. Thus, mice might be less suitable to study the mechanisms supporting vocal learning than previously assumed, despite their value for studying the genetic foundations of neurodevelopment more generally.

One of the central questions in human evolution is the origin of the human language faculty^1^,^2^. A key characteristic of human speech is that it is learned^1^. Comparative analyses of vocal learning in nonhuman animals have traditionally distinguished between production learning on the one hand and comprehension learning on the other. The former includes the modification of vocal output in response to auditory experience as well as the ability to use specific vocalizations in the appropriate situations^3^,^4^,^5^. The modification of vocal output encompasses the ability (and necessity) to imitate, such as in human speech acquisition, as well as more subtle forms of modification, such as vocal accommodation and the formation of dialects^6^. Among terrestrial mammals, humans appear to be unique in their ability to imitate sounds (including speech), although subtle forms of vocal modifications have been documented in a variety of species, and vocal learning has thus been conceived as a continuum rather than a discrete trait^7^. While humans share the basic neurological features subserving involuntary vocalizations with other terrestrial mammals^8^, the ability to voluntarily control vocal output and imitate auditory input is supported by a derived pathway connecting the posterior temporal cortex to the premotor cortex, most likely mediated via the parietal cortex^9^,^10^. Thus, in our species, cortical areas play a crucial role in auditory-to-motor mapping.

Recent years have seen an increasing interest in the effects of language-associated genes and their role in shaping the neural substrate of vocal communication. Of particular interest in this context are song birds, bats, and mice^11^,^12^,^13^,^14^,^15^. Because mice are more closely related to humans than birds or bats^16^, and because genetically modified models are readily available, they are particularly interesting to reconstruct the evolution of language related traits. Mice carrying the human variant of the *Foxp2* gene revealed slight differences in the structure of pup ultrasonic vocalizations (USVs), and increased synaptic plasticity and dendrite length in the medium spiny neurons in the striatum^17^. *SRPX2*, a target of FOXP2, was shown to modulate synapse formation, and a reduction of *SRPX2* led to a diminished number of USVs in mice^18^.

In support of the assumption that mouse vocalizations are at least partly shaped by experience, a developmental study revealed changes in the structure of USVs in relation to age and social context^19^, although it remained unclear whether this variation was due to cortical control of vocal production or to changes in motivation and/or maturation. More importantly, Arriaga and colleagues^20^ identified a weak direct cortical projection to brainstem vocal motor neurons, which may support volitional control of vocal output. They also deafened males at approximately 135 days by bilateral cochlear removal and compared the USVs of these males with age matched sham-operated males. Until the deafening procedure all male mice had developed their strain-specific USVs. After the procedures, deaf mice produced significantly more noisy USVs than their hearing littermates^20^.

Other studies failed to find evidence for effects of vocal learning. One such study made use of the fact that different genetic strains of mice differ in terms of their vocalizations. Male mice of two different genetic strains cross-fostered on the respective other strain, developed vocalizations typical for their genetic and not their foster parents^21^. Moreover, otoferlin-knockout mice, in which vesicle exocytosis in the inner hair cell is disrupted and which are profoundly deaf^22^,^23^ produced calls that did not differ from hearing wild-type littermates^24^, neither in terms of usage or structure. Similarly, adult male mice deafened at postnatal day 2 produced courtship vocalizations that did not differ from those of normal hearing animals^25^. All of the three studies strongly suggested that auditory feedback – a key feature of vocal learning – does not play an essential role in the development of strain-specific USVs.

To assess the importance of cortical structures for the development and production of mouse ultrasonic vocalizations more directly, we examined the vocal production of *Emx1-CRE*;*Esco2*^fl/fl^ mice, which are viable but lack the hippocampus and most of the cortex. Given the substantial evidence that mouse vocalizations are largely innate, the primary goal was to assess whether cortical projections have measurable effects on the usage and structure of mouse USVs. First, we compared the USVs of *Emx1-CRE*;*Esco2^fl/fl^* pups and control littermates during short term isolation at an age of 9 days. Second, we assessed the usage and structure of adult male USVs given in courtship encounters with females. If cortical structures have modulatory effects on the development of the usage and structure of vocalizations, *Emx1-CRE;Esco2^fl/fl^* subjects should differ in terms of the usage and/or structure of calls, compared to their control littermates.

## Results

**Figure 1:**
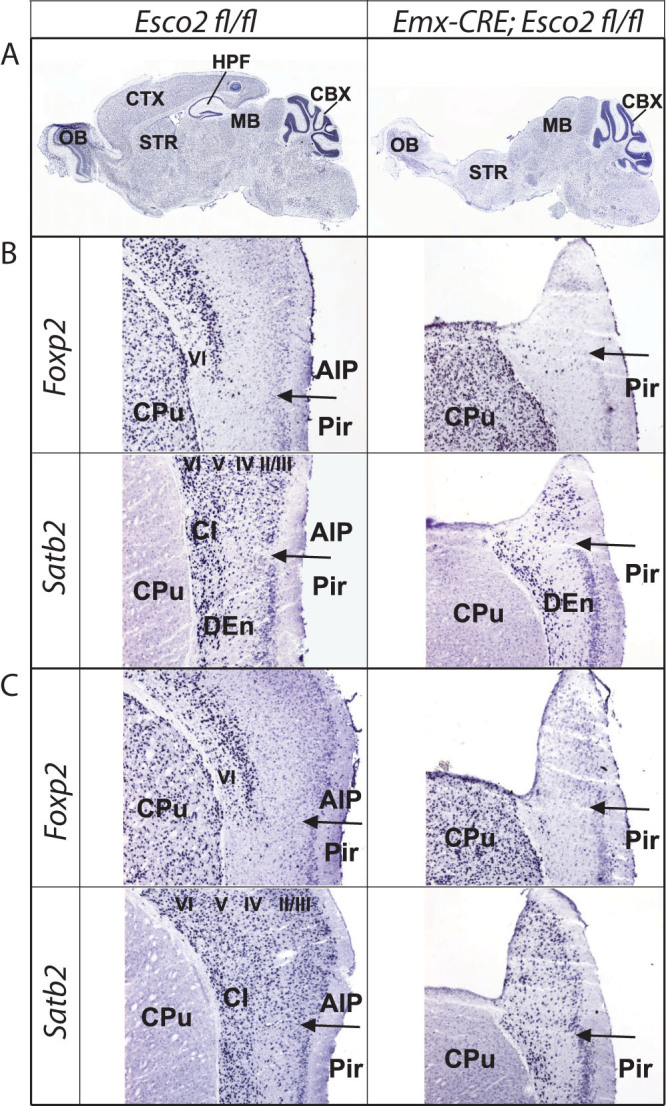
Anatomical and molecular characterization of cortical and hippocampal agenesis. (A) Nissl stained sections of control (*Esco2^fl/fl^*) and mutant (*Emx1-CRE;Esco2^fl/fl^*) at equal sagittal levels demonstrate the absence of cortical and hippocampal structures in *Emx1-CRE;Esco2^fl/fl^* animals. Abbreviations: CBX, cerebellum; CTX, cortex; HPF, hippocampus; MB, midbrain; OB, olfactory bulb; STR, striatum. (B) and (C): Expression analysis of *Foxb2* and *Satb2* in *Emx1-CRE*;*Esco2*^fl/fl^ and *Esco2^fl/fl^*mice. Sections are at Bregma levels 0.4 mm (B) and −0.7 (C). In controls, *Foxp2* transcripts are expressed in neocortical layer VI, while *Satb2* is expressed in all layers. In *Emx1^CRE^Esco2^fl/fl^* brains the ridge-like protrusion dorsal to the AIP/Pir boundary (marked by a horizontal arrow), does not contain *Foxp2*-expressing cells but the pan-layer marker *Satb2* is expressed. This suggests that these *Satb2*-positive neurons represent neurons with layer II to V characteristics. Abbreviations: AIP, agranular insular cortex; Cl, claustrum; CPu, caudate putamen; DEn, dorsal endopiriform nucleus;II-VI; cortical layers; Pir: piriform cortex.

*Emx1-CRE*;*Esco2*^fl/fl^ mice lack the hippocampus and most of the cortex (Fig. 1A). Morphological and histological analysis of the brain of *Emx1-CRE;Esco2^fl/fl^* mice showed that the piriform cortex was present in mutants. In addition, there is a small “ridge” protruding dorsally from the piriform cortex^26^. This ridge extends bilaterally and begins caudally of the olfactory bulb and ends at the midbrain (Fig. 1 B, C; right panels). To investigate whether this could be a residual neocortical structure, coronal sections were subjected to *in situ* hybridization with two neocortical markers *Satb2* and *Foxp2*^27^. In control brains, *Satb2* is expressed throughout the agranular insular cortex, while *Foxp2* mRNA is restricted to layer VI (Fig. 1 B, C; left panels). Subjecting coronal sections from mutant brains to *in situ* hybridization (Fig. 1 B, C; right panels) showed that *Foxp2* was not expressed in the ridge. By contrast, a small number of neurons positive for *Satb2* were observed. This suggests that the ridge-like structure dorsal of the piriform cortex seen in *Emx1-CRE;Esco2^fl/fl^* mice contains neurons that have layer II-V characteristics. None of these remaining cortical areas have been implied in auditory processing or motor control. In mammals, the brain pathway controlling innate vocalizations includes midbrain premotor structures and motoneuron pools in the medulla^7^. More specifically, limbic regions including the amygdala and the anterior cingulate cortex innervate the periaqueductal grey (PAG), which serves as a relay station. The PAG activates medullary premotor programs that eventually generate different acoustic patterns^7^.

**Figure 2:**
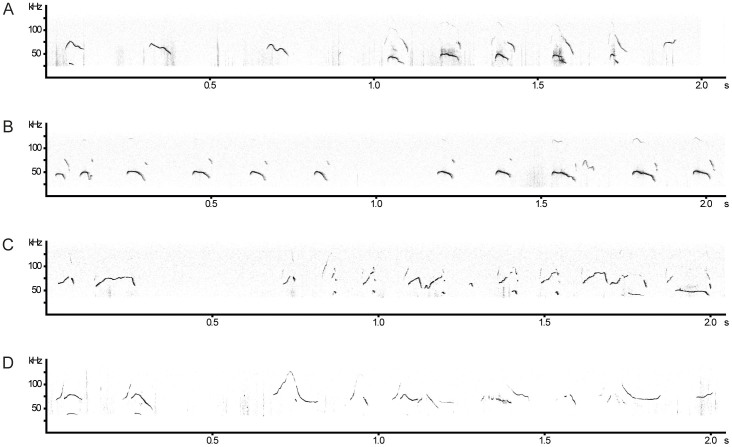
Examples of pup and male USVs. Despite substantial inter-individual differences we found no significant differences in the structure of the call sequence between *Emx1-CRE*;*Esco2*^fl/fl^ and control animals. (A): *Emx1-CRE*;*Esco2*^fl/fl^ pup, (B): control pup, (C): *Emx1-CRE*;*Esco2*^fl/fl^ male, (D): control male.

**Figure 3:**
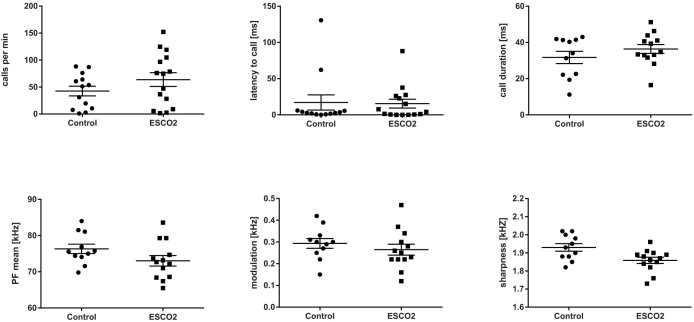
Number of calls/min, latency to call, and four frequency parameter for control and *Emx1-CRE*;*Esco2*^fl/fl^ pups. Marks represent the mean values of the individual subjects, lines indicate the mean ± SEM.

**Figure 4:**
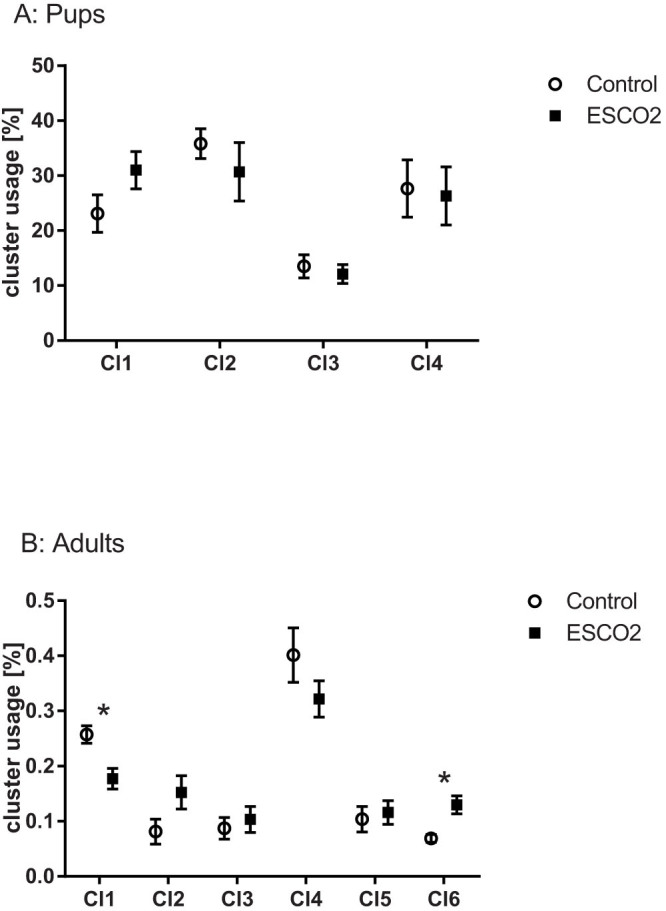
Percentage of cluster (call type) usage (mean ± SEM) for pups and adults. Stars indicate significant differences between conditions.

In the acoustic analysis, we first compared the USVs of 15 mutant (*Emx1-CRE*;*Esco2*^fl/fl^) pups and 13 control littermates (*Esco2*^fl/fl^) during isolation at an age of 9 d (Fig. 2). We found no significant difference in a suite of acoustic variables (Table 1) between mutant and control pups given during short-term isolation in the number of calls (Mann Whitney U-test: U = 120, *P* = 0.316, N_1_ = 15, N_2_ = 13, Fig. 3), the total amount of calling (U = 95, *P* = 0.186, N_1_ = 13, N_2_ = 11), the inter-call-interval (ICI) (U = 48, *P* = 0.173, N_1_ = 13, N_2_ = 11) or the latency to start calling (U = 94, *P* = 0.872, N_1_ = 15, N_2_ = 13, Fig. 3). A two-step cluster analysis revealed a cluster solution with 4 clusters as the best model. Cluster 1 (27.3% of all calls) comprised calls with a longer duration, the lowest start PF, the maximum PF in the last part of the call, and the highest positive frequency jumps. Cluster 2 (33%) contained short calls with the frequency maximum at the beginning of the call, and no frequency jumps. Cluster 3 (12.7%) contained the calls with the longest duration, a high maximum frequency at the beginning of the call and high negative frequency jumps. Cluster 4 (26.9%) comprised the shortest calls and only minor PF modulation without frequency jumps. The subsequent comparison between *Emx1-CRE*;*Esco2*^fl/fl^ and control mice revealed no significant differences in any of the acoustic variables (see Methods for specification, Fig. 3 and Table 2) or call type usage (Fig. 4A).

**Figure 5:**
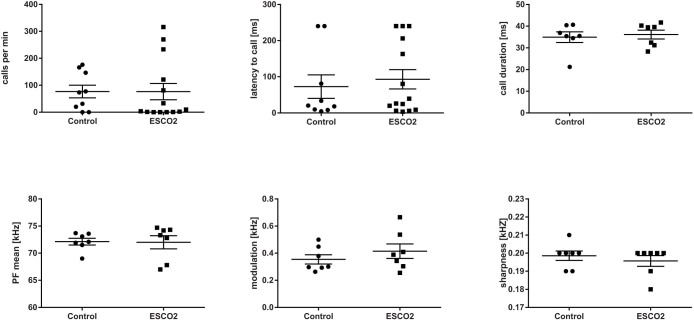
Number of calls/min, mean call duration latency to call, and four maximum peak frequency parameter and frequency slope for control and *Emx1-CRE;Esco^2fl/fl^* males. Marks represent the values of the individual subjects, lines indicate the mean ± SEM.

Next, we compared the structure and usage of USVs in adult males given during courtship encounters. We found no significant difference between 14 *Emx1-CRE*;*Esco2*^fl/fl^ and 9 control males in the number of calls (Mann Whitney U-test: U = 56, *P* = 0.688, N_1_ = 14, N_2_ = 9), the total amount of calling (U = 31, *P* = 0.779, N_1_ = 8, N_2_ = 7), the ICI (U = 24, *P* = 0.694, N_1_ = 8, N_2_ = 7), or the latency to start calling (U = 68, *P* = 0.781, N_1_ = 14, N_2_ = 9; Fig. 5). The two-step cluster analysis revealed a 6-cluster solution as the best solution, although the silhouette values differed only marginally between the 4-, 5-, and 6-cluster solution, indicating a relatively graded structure of the repertoire. Cluster 1 (21.7%) and cluster 6 (9.9%) both contained short calls. They differed with regard to the start and maximum PF: Cluster 1 had the maximum PF peak in the later part of the call, whereas in cluster 6, it was closer to the start of the call. Cluster 2 (11.7%) and cluster 4 (36.4%) contained calls of medium duration without major frequency jumps. Cluster 2 included calls with a high difference between start PF and maximum PF, whereas cluster 4 calls had low PF values. Cluster 3 (9.5%) was characterized by a relatively long call duration and the highest frequency jumps. Cluster 5 (11.8%) comprised the longest calls with medium frequency jumps (Table 3).

The comparison between *Emx1-CRE*;*Esco2*^fl/fl^ and control male USVs revealed no significant structural differences in any of the clusters (Fig. 5, Tab. 3). The same applied to the entire set of calls, without partitioning into call types. We found significant differences only in the usage of call types. *Emx1-CRE*;*Esco2*^fl/fl^ male mice used calls from cluster 1 more frequently and calls from cluster 6 less frequently than the controls (Fig. 4B). Both clusters comprised calls with a short duration without major frequency jumps. Cluster 6 had a higher start frequency and a steeper negative slope, whereas cluster 1 had only a minor negative slope (see Table 3). Note however that both *Emx1-CRE*;*Esco2*^fl/fl^ and control mice were able to produce calls from all clusters.

## Discussion

The acoustic analysis did not reveal significant differences in the acoustic structure of *Emx1-CRE;Esco2^fl/fl^* mice and their control littermates. The results indicate that in mice, the cortical areas lacking in *Emx1-CRE;Esco2^fl/fl^* mice are not necessary to develop the vocal structure or usage typical for the strain^21^,^28^. As mice start to hear at an age of app. 10-12 days^29^ and an immediate response to isolation is important for the survival during the first days of life, the lack of a difference in both usage and structure of pup USVs is perhaps not so surprising. In contrast, we deemed it more likely that the complex adult male songs were to some degree under cortical control, such that *Emx1-CRE*;*Esco2*^fl/fl^ males would respond in a diminished or aberrant fashion compared to their control littermates. This was not the case. Although we cannot rule out that a much larger sample size would reveal very small effects, we believe the distribution of the values (Fig. 5) supports the view that there are no substantial differences in the acoustic structure between the two groups. One exception is perhaps the number of calls, where *Emx1-CRE;Esco^2fl/fl^* males appear to reveal a higher proportion of extreme values, with some subjects calling more frequently and others less frequently than control subjects. This may be due to the fact that cortical areas play a role in generating and controlling motivational tendencies in courtship behavior. One may question whether the lack of modification is restricted to vocalizations uttered in courtship encounters. We focused on this context for two reasons: firstly, encounters with females most reliably elicit calls from males; secondly, this context involves a number of different social behaviors, and the vocalizations in this context are considered to be the most complex utterances in this species^30^,^31^,^32^. As cortical control seems more likely in more complex utterances than simpler ones, we would expect to see a potential difference primarily in the more complex calls. Notably, we also found no differences in the temporal patterning of male songs, implying that the lack of the cortical structures does not affect this feature either. Whether the call amplitude was affected remains unclear, since the setting did not allow us to collect reliable amplitude measurements.

Our findings thus strongly suggest that both mouse pup isolation calls and male courtship vocalizations constitute basal behavior patterns supported by evolutionary older encephalic structures, including the striatum and the midbrain. Apparently, these remaining structures were sufficient to perceive the isolation and mating situation as such, and to initiate the appropriate behavior, including the production of vocalizations that did not differ significantly from control subjects. Nevertheless, these findings do not rule out the possibility that the mouse cortex is important for processing and integrating information from different sensory domains, as well as learning and memory, which all contribute to the regulation of behavior at a more fine-grained level. Thus, it may be the case that the weak direct cortical projection to brainstem vocal motor neurons identified by Arriaga and colleagues^7^,^20^ have some function in the modulation of the vocal output, although this projection is clearly not necessary to generate the key behavioral patterns. Conceptually, it is therefore crucial to distinguish between obligate and facultative learning; mice (or the majority of terrestrial mammals studied to date) are obviously not obligate learners, and vocal production appears to be largely robust, despite the fact that large parts of the brain are essentially missing. The present results are relevant for studies that investigated the effects of language-associated genes on the vocal output of the respective mouse models. Because evidence is accumulating that the mechanisms supporting mouse vocal behavior and human speech are fundamentally different, our findings indicate that mice might be less suitable to study the mechanisms supporting vocal learning than previously assumed. Investigations of the motivational components underpinning communicative behavior thus appear much more promising^15^. In addition, the question whether and in which way auditory experience may shape vocal output at a small scale, resulting in minor but perhaps meaningful modifications, deserves further attention^6^.

## Methods

### Subjects

Breeding and genotyping of animals was carried as described previously^26^. Mice were housed in polysulfon cages, covered by wire lids with food and water bottles in a pathogen-free area. Cages were supplied with filtered air and contained nesting material. All experiments were performed in accordance with relevant guidelines and regulations. All methods were approved by the Lower Saxony State Office for Consumer Protection and Food Safety (Document Number: 33.11.42502-04-095/07).

### Histological analysis and in situ hybridisation

Nissl staining and robotic in situ hybridization on paraformaldehyde fixed sections of 6-weeks old brains was performed as described previously^33^ using the probes whose sequence can be retrieved from www.genepaint.org under the following Set IDs: EG742 (Foxp2) and EG1239 (Satb2).

### Recordings and Acoustic Analysis

For the isolation test pups were selected randomly from their litter, weighed and placed in a soundproofed custom made plastic box (diameter 13.5 cm). An ultrasound microphone (UltraSoundGate CM16) fixed in the lid of the box 12 cm above the bottom was connected to a preamplifier (UltraSoundGate 116), which was connected to a notebook computer. In total we tested 28 pups at an age of 9 days, 15 *Emx1-CRE*;*Esco2*^fl/fl^ and 13 control littermates. The recording duration of a single session was 4 minutes.

To test the males in the courtship design each male was separated in a single macrolon 2 cage (36.5 × 21 × 14 cm) one day before the test. For the recordings, the cages with the males were placed in a sound-attenuated Styrofoam box and after three minutes, a female (*Emx1-CRE*;*Esco2*^fl/fl^) was introduced in the male cage for four minutes. In total we tested 23 adult males (age of 6 weeks), 14 *Emx1-CRE*;*Esco2*^fl/fl^ and 9 control littermates.

The sampling frequency of 300 kHz resulted in a frequency range of 150 kHz. We used the whistle tracking algorithm of Avisoft-SAS Lab Pro 5.2 (R. Specht, Berlin, Germany) with following settings: monotonic, maximum change per step 8 pix = 4.7 kHz, minimum continuity = 5 ms (pups), 8 ms (adults), hold time = 15 ms. Because sound energy outside the frequency range of the produced USVs can have a negative influence on the estimations, we applied a high pass FIR filter of 35 kHz. These criteria were compared with former analysis of pup and male mouse vocalizations^24^,^31^,^34^. Based on these settings we calculated the following parameters: number of given calls, ICI (inter call interval measured from the end of a call to the start of the next call), and latency to start calling (time from placing the pup into the box until first call, or time from placing the female into the box with the male, until first call). In addition we check visually the outcome of the automatic procedure because in rare cases (in our study: 3.5% pups, 1.2% adults) the program can select other sounds such as toe clicking, sniffing or high frequency background erroneously as USVs.

We used the same algorithm to cut out the single ultrasounds and stored them as single wave files. From the stored calls, we calculated high-resolution spectrograms (frequency range: 150 kHz, frequency resolution: 293 Hz, time resolution: 0.21 ms) and submitted the resulting spectrograms to the custom software program LMA 2013^35^ to extract a set of characteristic acoustic parameters. As mice typically concentrate the energy of their calls into one small frequency band, so-called “whistles” or “pure tone-like sounds” (see Fig. 2) we focused on the peak frequency, i.e. the loudest frequency in the spectrum, which corresponds in most cases to the fundamental frequency (F0). Mice often produce soft sounds and just small head movements can lead to strong amplitude fluctuations in USVs. Therefore, we visually controlled the estimation of acoustic parameters and excluded incorrect estimated calls from the analysis.

For each call we determined the duration of a call and the duration of amplitude gaps within a call. We defined the start of a call when the sound energy of a time segment is above 10% of the mean maximum amplitude of this call. An amplitude gap is defined if the sound energy of a certain time segment goes below 10%. To determine the end of a call we used the same threshold (10%). In addition, we calculated start, maximum and mean peak frequency, the sharpness of the frequency peak and the greatest difference in peak frequency between two consecutive 0.21 ms bins (so-called frequency jumps, a characteristic feature in mouse ultrasound (e.g. Ref. 30). For further characterization of call modulation we calculated the location of the maximum frequency, the slope of a linear trend and the modulation of peak frequency (for further details see Tab. 1). For the subsequent statistical tests we selected a balanced selection of 2180 pup isolation calls and 1835 adult male courtship vocalizations of sufficient quality, taken from all subjects that produced vocalizations.

### Statistics

Because an analysis of the unpartitioned data set, including all calls, may cover subtle acoustic differences, we partitioned the data set into different call types using a two-step cluster analysis (CA, IBM SPSS 21). As pup isolation calls and male courtship vocalization differ in structure, we calculated separate cluster solutions for pup and adult male vocalizations. We used the log-likelihood function as distance measure because this measure is less susceptible against outliers, and the Schwarz'sches Bayes Criterion (BIC) to find the best cluster solution. We used the seven acoustic parameters indicated in Table 1 for the CA. Using a higher number of parameters usually provides no advantage, because highly correlating acoustic parameters render it difficult to find appropriate cluster centers^24^. We assessed the quality of the cluster solutions by calculating the silhouette values, which represent a measure of the distinctiveness between clusters. Silhouette coefficients (S_c_) may range between −1.0 and 1.0 and values > 0.5 are usually considered as solid solutions^36^. Because mice have a relative graded vocal repertoire^21^,^24^,^25^,^31^, we accepted cluster solutions with an S_c_ > 0.3 and selected the solution with the highest number of clusters for further statistical testing.

To test differences in call number, ICI, latency to start calling and cluster usage we used exact Mann Whitney U-test. In all other tests in which we have multiple calls per subject we used a linear mixed model with genotype as fixed factor and subject as random factor. All tests we done with IBM SPSS 21. Whenever necessary, we corrected p-values for multiple testing using Simes correction. The Simes correction is a correction methods for multiple testing which minimize the β error.

## Author Contributions

J.F., K.H. and G.E. conceived the experiment, K.H. and G.W. carried it out and conducted the data analysis, and J.F. wrote the paper with input from all authors.

## Figures and Tables

**Table 1:** Description of acoustic variables used in the analysis. Asterisks mark the acoustic variables used to estimate the vocal clusters

Acoustic feature	Description
Duration [ms] *	Time between onset and offset of call
Amplitude gap [ms] *	Duration of breaks in amplitude within call
PF start [Hz] *	Start frequency of peak frequency
PF mean [Hz]	Mean frequency of peak frequency
PF max [Hz] *	Maximum peak frequency
PF max loc *	Location of PF max in relation to total call duration [(1/duration) * loc]
PF jump [Hz] *	Maximum difference of peak frequency between successive bins
PF modulation [Hz]	Mean frequency differences between original PF and floating average
Slope of trend *	Factor of the linear trend of the peak frequency through the peak frequencies of consecutive 0.21 ms bins
PF sharpness [Hz]	Width of PF peak at 25% amplitude below the maximum amplitude peak

**Table 2:** Acoustic features of control and *Emx1-CRE*;*Esco2*^fl/fl^ (ESCO2) pups (mean ± SEM), for the unpartioned data set (all calls) and separately for the 4 clusters

Acoustic feature	Genotype	All	Cluster1	Cluster2	Cluster3	Cluster4
Duration [ms]	control	35.5 ± 0.7	43.6 ± 1.2	28.6 ± 0.8	53.8 ± 1.6	23.1 ± 1.1
	ESCO2	38.2 ± 0.6	45.6 ± 0.8	29.2 ± 0.7	60.6 ± 2.1	29.0 ± 0.8
Ampl. gap [ms]	control	2.4 ± 0.2	3.6 ± 0.3	0.5 ± 0.1	7.6 ± 0.7	1.0 ± 0.2
	ESCO2	3.0 ± 0.2	4.0 ± 0.3	0.5 ± 0.1	11.3 ± 1	0.7 ± 0.1
PF start [kHz]	control	75.0 ± 0.5	60.0 ± 0.6	87.8 ± 0.5	84.5 ± 1.5	63.8 ±0.4
	ESCO2	72.0 ± 0.4	60.8 ± 0.3	86.5 ± 0.4	79.8 ± 1.5	62 ± 0.4
PF mean [kHz]	control	77.2 ± 0.4	69.5 ± 0.6	89.4 ± 0.4	77.5 ± 1	65.5 ± 0.4
	ESCO2	73.2 ± 0.3	66.7 ± 0.3	86.2 ± 0.3	73.8 ± 0.9	62.6 ± 0.4
PF max [kHz]	control	89.0 ± 0.5	94.5 ± 0.5	95.2 ± 0.5	99.8 ± 1.8	68.1 ± 0.4
	ESCO2	86.2 ± 0.4	91.9 ± 0.4	99.8 ± 1.1	95.7 ± 1.3	65.2 ± 0.4
PF max loc	control	0.46 ± 0.01	0.72 ± 0.02	0.35 ± 0.01	0.3 ± 0.02	0.48 ± 0.02
	ESCO2	0.45 ± 0.01	0.75 ± 0.01	0.28 ± 0.01	0.3 ± 0.02	0.34 ± 0.02
PF jump [kHz]	control	5.1 ± 0.8	39.8 ± 0.6	0.2 ± 0.4	−29.1 ± 2.6	1.2 ± 0.2
	ESCO2	9.3 ± 0.7	37.7 ± 0.4	−0.4 ± 0.3	−19.7 ± 2.7	0.0 ± 0.2
PF modulation [Hz]	control	292 ± 10	469 ± 22	173 ± 8	613 ± 38	111 ± 6
	ESCO2	267 ± 9	419 ± 17	144 ± 6	541 ± 44	89 ± 3
Slope of trend	control	4.6 ± 0.1	2.7 ± 0.2	4.8 ± 0.2	2.3 ± 0.1	7.3 ± 0.4
	ESCO2	3.9 ± 0.1	2.3 ± 0.08	4.8 ± 0.2	2.4 ± 0.1	5.8 ± 0.3
PF sharpness [Hz]	control	1926 ± 8	1886 ± 14	2009 ± 15	1928 ± 16	1837 ± 16
	ESCO2	1863 ± 6	1841 ± 7	1945 ± 11	1881 ± 13	1762 ± 10

**Table 3:** Acoustic features of vocalizations from control and *Emx1-CRE*;*Esco2*^fl/fl^ (ESCO2) males (mean ± SEM), for the unpartitioned data set (all calls), and separately for the six clusters

Acoustic feature	Genotype	All	Cluster1	Cluster2	Cluster3	Cluster4	Cluster5	Cluster6
Duration [ms]	control	36.6 ± 0.8	29.1 ± 0.8	34.6 ± 1.5	39.9 ± 1.7	31.4 ± 0.1	76.1 ± 3	30.6 ± 2.1
	ESCO2	37.9 ± 0.7	29.7 ± 0.1	39.3 ± 1	39.1 ± 1.4	30.8 ± 1.0	72.3 ± 2.7	32.7 ± 1.5
Ampl. gap [ms]	control	2.0 ± 0.1	0.7 ± 0.1	2.1 ± 0.3	1.8 ± 0.3	0.7 ± 0.1	10.6 ± 0.7	1.1 ± 0.3
	ESCO2	1.9 ± 0.1	0.5 ± 0.1	1.5 ± 0.2	1.6 ± 0.2	0.4 ± 0.1	10.9 ± 0.6	0.8 ± 0.1
PF start [kHz]	control	73.2 ± 0.4	67.8 ± 0.5	72.5 ± 1.2	65.7 ± 1.4	75.0 ±0.3	71.7 ± 0.8	98.7 ± 1.9
	ESCO2	74.6 ± 0.5	66.8 ± 0.6	74.1 ± 0.9	64.6 ± 1.3	73.9 ± 0.4	72.2 ± 1.2	98.5 ± 1.2
PF mean [kHz]	control	71.8 ± 0.2	72.6 ± 0.4	75.2 ± 0.9	71 ± 1	70.1 ± 0.3	70.1 ± 0.7	77.2 ± 1.2
	ESCO2	71.8 ± 0.4	70.7 ± 0.7	7.7 ± 0.1	72.3 ± 1.2	67.8 ± 0.4	70.3 ± 1	76.7 ± 1.1
PF max [kHz]	control	85.2 ± 0.5	79.1 ± 0.5	99.0 ± 1.7	102.6 ± 2	77.6 ± 0.3	87.4 ± 1.5	102 ± 1.9
	ESCO2	89.1 ± 0.6	76.7 ± 0.8	101.4 ± 1.2	103.2 ± 1.5	75.9 ± 0.4	93.5 ± 1.8	101.9 ± 1.2
PF max loc	control	0.41 ± 0.01	0.71 ± 0.01	0.66 ± 0.02	0.68 ± 0.03	0.1 ± 0.01	0.48 ± 0.03	0.07 ± 0.02
	ESCO2	0.41 ± 0.01	0.72 ± 0.01	0.68 ± 0.02	0.66 ± 0.03	0.09 ± 0.01	0.49 ± 0.03	0.08 ± 0.01
PF jump [kHz]	control	1.3 ± 0.8	4.0 ± 0.7	−3.9 ±2.1	43.4 ± 1.6	−1.6 ± 0.5	11.0 ± 2.9	−2.4 ± 2.7
	ESCO2	−4.2 ± 0.9	−0.3 ± 0.8	−3.5 ± 1.4	40.7 ± 1.5	−3.2 ± 0.6	7.5 ± 3.2	−2.6 ± 1.6
PF modulation [Hz]	control	372 ± 14	220 ± 10	845 ± 78	898 ± 58	197 ± 7	337 ± 31	555 ± 49
	ESCO2	461 ± 16	192 ± 15	847 ± 56	857 ± 48	199 ± 9	431 ± 42	544 ± 33
Slope of trend	control	−0.1 ± 0.01	0.2 ± 0.01	0.0 ± 0.04	0.2 ± 0.05	−0.3 ± 0.01	−0.1 ± 0.02	−0.6 ± 0.09
	ESCO2	−0.1 ± 0.02	0.2 ± 0.02	0.1 ± 0.03	0.3 ± 0.05	−0.4 ± 0.02	−0.1 ± 0.02	−0.6 ± 0.06
PF sharpness [Hz]	control	1996 ± 7	1978 ± 14	2048 ± 22	2032 ± 22	2004 ± 12	1904 ± 17	2045 ± 29
	ESCO2	1956 ± 7	1880 ± 13	2019 ± 19	2008 ± 22	1939 ± 14	1899 ± 19	2009 ± 21
